# Supplementation with açaí (Euterpe Oleracea Martius) for the treatment of chronic tinnitus: effects on perception, anxiety levels and oxidative metabolism biomarkers

**DOI:** 10.1590/2317-1782/20212021076

**Published:** 2022-01-26

**Authors:** Sheila Jacques Oppitz, Michele Vargas Garcia, Rúbia Soares Bruno, Cleide Monteiro Zemolin, Bruna Olegário Baptista, Bárbara Osmarin Turra, Fernanda Barbisan, Ivana Beatrice Mânica da Cruz, Aron Ferreira da Silveira

**Affiliations:** 1 Programa de Pós-graduação em Distúrbios da Comunicação Humana, Universidade Federal de Santa Maria – UFSM - Santa Maria (RS), Brasil.; 2 Programa de Pós-graduação em Gerontologia, Universidade Federal de Santa Maria – UFSM - Santa Maria (RS), Brasil.; 3 Hospital Universitário de Santa Maria, Universidade Federal de Santa Maria – UFSM - Santa Maria (RS), Brasil.; 4 Departamento de Morfologia, Universidade Federal de Santa Maria – UFSM - Santa Maria (RS), Brasil.

**Keywords:** Hearing, Tinnitus, Anxiety, Euterpe, Antioxidants, Oxidative Stress, Placebo Effect

## Abstract

**Purpose:**

To investigate the effects of antioxidant supplementation with açaí extract on the discomfort with chronic tinnitus and the relationship with the levels of anxiety and oxidative metabolism, not excluding the overlap of diseases.

**Methods:**

Randomized, placebo-controlled clinical trial. 30 individuals participated, with an average of 50.5 years, 14 males and 16 females, with normal hearing thresholds or sensorineural hearing loss up to mild degree, divided into two groups: Placebo Group (without active) and, Açaí Group (100mg of açaí extract). The following procedures were applied before and after three months of treatments: Tinnitus Handicap Inventory (THI), Beck's Anxiety Inventory (BAI) and blood samples for evaluation of oxidative stress biomarkers (Lipid Peroxidation and Protein Carbonylation).

**Results:**

There was a reduction in the discomfort of tinnitus for the açaí group verified through THI (p = 0.006). Significant differences were found in the score of common symptoms for anxiety disorders in the placebo group (p = 0.016), however, the same was not observed for oxidative metabolism biomarkers, although there was a decrease in post-treatment values for all groups.

**Conclusion:**

Oral antioxidant supplementation, with açaí extract, showed favorable effects on tinnitus, reducing discomfort with the symptom, regardless of the underlying etiology, and can be considered a treatment modality. However, the effect of this supplementation on anxiety symptoms and oxidative stress biomarkers needs further investigation.

## INTRODUCTION

Açaí (Euterpe oleracea Mart.) is a typical fruit from northern Brazil which was recently popularized for its high antioxidant capacity related to the presence of phenolic acids, flavonoids and anthocyanins^(1)^. Currently, different studies have been carried out with this fruit, such as in cases of diabetes, dyslipidemia, cardiovascular disease, antihypertensive effect^(2)^, alternative therapy for neuropsychiatric diseases^(3)^, among others.

Antioxidants are compounds that act by inhibiting and/or decreasing the effects of oxidative stress, triggered by free radicals and oxidizing compounds. They are important because, with the combat against oxidative processes, there is less damage to Deoxyribonucleic Acid (DNA) and also to macromolecules, thus alleviating the cumulative damage that can trigger different diseases^(4)^.

The nature of the cell damage caused by Reactive Oxygen Species (ROS) depends on its place of formation, however, the main molecules susceptible to damage induced by oxidative stress are membrane lipids, where there is destruction of their integrity, causing death or cell apoptosis. When there is an increase in the formation of ROS in the Organ of Corti, it results in permanent sensory epithelial damage^(5)^.

In relation to tinnitus, there are reports of higher plasma concentrations in oxidative stress biomarkers and low antioxidant activity, supporting the role of oxidative status in the pathogenesis of tinnitus. However, the use of oxidative stress biomarkers for comparing before and after treatments are scarce, current studies with biomarkers show symptomatological improvement, but little or no difference in oxidative status^(6,7)^.

According to the literature, antioxidant therapy in patients with idiopathic tinnitus can reduce oxidative stress and damage to inner ear, also reducing tinnitus intensity and discomfort. Antioxidant supplementation may be an additional therapy option for patients with tinnitus, however, clinical studies are necessary to determine the protective role of antioxidants and choose the most appropriate therapeutic protocol^(8)^.

Tinnitus is considered a health problem that has an economic impact. A survey showed that health expenses for patients with tinnitus complaints are high, as, in general, this group seeks assistance from several professionals. In addition, the authors highlighted that health care costs are directly proportional to the severity of the symptom^(9)^.

Currently, there is no effective treatment for all patients. The heterogeneity of tinnitus is an additional challenge to clinical research, as the symptom can differ in many aspects, such as location, sound features, underlying causes, comorbid conditions, among others^(10)^.

Although there are advances in specific studies on tinnitus, its physiopathology and, consequently, etiology have not yet been completely clarified. Allied to the subjectivity of this manifestation, plus the overlapping of illnesses and symptoms that usually affect these patients, makes it difficult to obtain a good therapeutic result^(11)^. However, seeking new measures that address the individual as a whole and help in reducing tinnitus becomes necessary. It should also be noted that, due to the multifactorial nature of the symptom, the treatment often needs to be combined and not isolated.

Then, the açaí extract has an attractive pharmacological, antineoplastic and antioxidant profile with properties without side effects, which suggests its use in prevention therapy or treatment of symptoms and diseases^(12)^.

Therefore, natural foods, such as açaí extract, could help subjects with chronic tinnitus to reduce the discomfort with the symptom. Thus, the aim of this study was to investigate the effects of antioxidant supplementation with açaí extract regarding the discomfort with chronic tinnitus and the relationship with levels of anxiety and oxidative metabolism, not excluding the overlapping of illnesses.

## METHODS

Double-blind study, with a qualitative and quantitative approach, developed in the form of a randomized clinical trial, with a descriptive feature, with a transversal and longitudinal design. The sample consisted of convenience and the auditory care were carried out in an Audiology Outpatient Clinic of a Public Institution of Higher Education.

### Ethical aspects

Approved by the Research Ethics Committee under number CAAE: 96740718.4.0000.5346, with the Brazilian Registry of Clinical Trials (ReBEC) number: RBR-8z4mhq. Individuals who agreed to participate in this research signed the Free and Informed Consent Term drawn up in accordance with Resolution 466/12, of the National Health Council.

### Eligibility criteria

To participate in the study, individuals must be 18 years or older; Complaint of chronic tinnitus (minimum perception of six months) unilateral or bilateral; Hearing thresholds within normal ranges bilaterally^(13)^ or sensorineural hearing loss from a degree to mild in the quadritonal mean (500, 1000, 2000 and 4000Hz)^(13)^; Annoyance score of at least four on the Visual Analog Scale, considered a moderate annoyance of the symptom^(14)^.

In addition, they could not, during the period of participation in this study, have presented symptoms and/or diagnosis of middle ear involvement, have started a new treatment (pharmacological or therapeutic) or have been diagnosed with any disease of any origin. They could not make use of individual sound amplification devices (ISAD) in order that no interference would occur in the findings of the present study.

### Sample characterization

Initially, 62 individuals were invited to participate in this study, and of these, eight did not attend the auditory care, five had already undergone previous treatment for the symptom and five had sensorineural hearing loss of degree greater than moderate. In addition, four reported a Visual Analog Scale (VAS) score of less than three, one was pregnant, nine did not complete the three months of treatment, being a total of 30 volunteers in the sample, 15 in each group, with a mean age of 50.5 years, 14 males and 16 females.

The individuals were divided into two different groups and received one of the following therapeutic schemes: The placebo was the control group, without active, available through starch capsules and the açaí extract was the group that received the treatment with administration of extract of dry açaí (100mg/capsule).

Randomization was performed in order that each research volunteer could have the same probability of being allocated to the two study groups, contributing to homogeneous sample features. It was carried out in the form of drawing cards, in which the volunteer removed the card corresponding to one of the proposed treatments.

The sample calculation was based on the variation of the total score of THI questionnaire, adopting a significance level of 5% and a test power of 80%, for detecting a minimum difference between the participants of 20 points in the total score of the questionnaire, it was estimated that at least 15 subjects were needed, according to the One-way ANOVA software.

In relation to the procedures which were performed, the following detailing is described:

**Tinnitus Investigation Anamnesis:** Anamnesis based on the Clinical Practice Guideline: Tinnitus^(15)^, containing information about clinical and medical history, habits, and specific questions about tinnitus (location, frequency, intensity, time of perception, among others). It is importante to note that all comorbidities were considered through the report of the individual.
**Tinnitus Handicap Inventory – THI:** Instrument translated and validated for Brazilian Portuguese, consisting of 25 questions and three options of answes: yes (4 points), sometimes (2 points) and no (0 points). The score can range from 0 (zero) to 100 points. Thus, scores between 0 and 16 indicate a degree of mild annoyance, between 18 and 36, mild annoyance, between 38 and 56, moderate, between 58 and 76, intense annoyance, and between 78 and 100, catastrophic annoyance. The questionnaire was applied in the form of an individual interview in order to ensure full completion and understanding of the question by the researched individual^(16)^.
**Beck Anxiety Inventory – BAI:** This inventory was adapted by Cunha (2001)^(17)^, presenting good reliability and validity coefficients. The scale consists of 21 items describing common symptoms of anxiety, the answer is within a four-point scale: Absolutely not; Slightly; Moderately; Seriously. The added items result in a total score that could range from zero to 63, generating the level of anxiety: 0 – 7 points = minimum level of anxiety; 8 – 15 points = mild anxiety; 16-25 points = moderate anxiety; 26-63 points = severe anxiety.
**Assessment of oxidative metabolism:** After the completion of the auditory assessments, peripheral blood samples (20mL) were collected from the arm of a volunteer, using Vacutainer ® top tubes (BD Diagnostics, Plymouth, United Kingdom), with Ethylenediaminetetraacetic Acid (EDTA), an anticoagulant. The collection was carried out in the initial and final evaluation by a professional nurse with experience in blood.

Initially, the samples were centrifuged at 2000 rotations per minute (rpm) for 10 minutes, in order to separate the plasma, white blood cells and erythrocytes. The white blood series was placed in tubes with Ficoll-Histopaque-Sigma (Ficoll-Paque Plus - GE HealthCare, density 1.077), occuring the centrifugation, separation, washing and placed in tubes with trizol for further analysis. The plasma was placed in microtubes and stored at -80ºC for further assessments.

Oxidative stress biomarkers do not present normality criteria in the literature, what is known is that the higher the values, the greater the damage, therefore, individuals should be self-compared before and after treatment. With the plasma, the following analyzes were performed:

Lipid Peroxidation (Lipoperoxidation) - TBARS Assay (Thiobarbituric Acid Reactive Substances): Lipoperoxidation (LPO) can be defined as a cascade of biochemical events resulting from the action of Free Radicals (FR) on unsaturated lipids in cell membranes, leading to severe alteration of the cell membrane, causing loss of fluidity, alteration of the secretory function and transmembrane ionic gradients. Alterations in membranes lead to permeability disorders, altering the ionic flux and the flux of other substances, which results in the loss of selectivity for the entry and/or exit of nutrients and substances which are toxic to the cell, changes in DNA (Deoxyribonucleic Acid) and impairment of extracellular matrix components (proteoglycans, collagen and elastin).

Therefore, lipid peroxidation was evaluated with the use of TBARS assay through the reaction of thiobarbituric acid with Malondialdehyde (MDA), the greater the presence of MDA, the greater the lipoperoxidation indexes. All treatments were added with water, phosphoric acid (H_3_PO_4_) to 10%, trichloroacetic acid (TCA) to 20% and thiobarbituric acid (TBA) to 1.2%. To this end, a dosage curve of malondialdehyde (MDA) for further determination of the equivalence of each test sample. After adding all reagents, an incubation was carried out for one hour at 95ºC, and then, with the samples at room temperature, the absorbance reading at 532 nm was performed in a spectrophotometer. Results were expressed as nmol of MDA/mL of cells^(18)^.

Protein Carbonylation: During oxidative stress in proteins, it occurs the chain fragmentation and oxidation of almost all types of amino acids with frequent production of carbonyl compounds such as aldehydes, ketones, amides, carboxyls and esters. These damages were measured by determining the formation of carbonyl groups based on the reaction with 2,4-dinitrophenyl-hydrazine (DNPH), a reagent that reacts with carbonylated proteins as previously described by Morabito et al. (2004)^(19)^. The carbonyl formation content is determined by reading in a plate reader, in an ELISA UV plate at a wavelength of 370nm.

Therefore, for the primary outcome, it is necessary to observe the improvement of tinnitus annoyance in the lives of individuals through the decrease in THI scores, so that the secondary outcome is also based on the decrease in scores, but also for anxiety (BAI) and oxidative metabolism biomarkers (TBARS and carbonylation).

### Açaí extract (Euterpe Oleracea Martius) as a form of experimental treatment

The açaí extract was supplied through properly patented capsules obtained by the company PHARMANOSTRA, with a certificate of analysis and registration by the National Health Surveillance Agency (ANVISA) nº 669820036. The chemical and pharmacological features were preserved, guaranteeing its biological action, the safety of use and the enhancement of its therapeutic potential.

In relation to the dosage of açaí extract, it was used 100mg a day (one capsule a day) of the fruit pulp. A pilot study tested the dosage by comparing 50mg of açaí in capsules; 100mg; and 500mg or more (self-medication), being more effective with 100mg. Subjects who self-medicated with 500mg or more had a daily moderate tension headache as an adverse effect that, within two days after the decrease to 100 milligrams, the headache disappeared, but the tinnitus continued. Therefore, the dosage of açaí needs to be carefully and periodically monitored, in order to avoid mistaken self-medication, with a limit of the extract that defines it as natural or toxic to the body, which may result in tension headache of moderate intensity and increased tinnitus perception.

All volunteers were instructed to take one capsule a day, at the same time, preferably with water, for three months, a minimum duration for significant changes to occur in relation to oxidative metabolism. The individuals returned monthly to remove the capsules with the researcher. The substances used in the treatment were not identified by name in the bottles in which they were stored, but with symbols defined by an external individual, as a way of blinding researchers and volunteers.

It is noteworthy that, in the present study, there was no occurrence of side effects or adverse effects with the use of substances during the time and regarding the dose which was used (100mg). Being that, one of the relevant factors to be taken into consideration during a treatment.

After the experimental treatment, the individuals were submitted to a reassessment composed by the same procedures of the first assessment, previously listed. In addition, Speech Therapy Counseling was offered after the reassessment, as well as referrals to professionals when necessary.

Data were registered in Microsoft Office Excel (2010) and the distribution of normality was determined by the Kolmogorov-Smirnov parametric test. To calculate hypotheses for independent nominal variables, the Chi-Square test was used. Normally distributed variables were evaluated using the Kruskal-Wallis test. The accepted level of significance was p<0.05 (5%) with confidence intervals of 95%.

## RESULTS

Regarding the features of the sample, in relation to hearing, the sensorineural hearing loss in high frequencies (55.6%), hearing thresholds within normal standards (33.3%) and mild sensorineural hearing loss (11.1%) were the most prevalente ones.

In relation to tinnitus, the most common type was buzzing with continuous manifestation, the predominant psychoacoustic measures were: frequency of 6000 Hz and intensity of 15 dB.

The presence of some comorbidities may be related to the discomfort and permanence of tinnitus (Table 1).

**Table 1:** Comparison of comorbidities between groups

		**Açaí**		**Placebo**		**Total**		**P-value^1^**
		N	%	N	%	N	%	
TMD	No	10	66.7%	7	46.7%	17	56.7%	0.293
	Yes	5	33.3%	8	53.3%	13	43.3%	
Dizziness	No	10	66.7%	14	93.3%	24	80%	0.146
	Yes	5	33.3%	1	6.7%	6	20%	
Psychological	No	6	40.0%	5	33.3%	11	36.7%	0.741
	Yes	9	60.0%	10	66.7%	19	63.3%	
Hypercholesterolemia	No	11	73.3%	11	73.3%	22	73.3%	0.887
	Yes	4	26.7%	4	26.7%	8	26.7%	
Hypertension	No	10	66.7%	12	80.0%	22	73.3%	0.484
	Yes	5	33.3%	3	20.0%	8	26.7%	
Hormonal	No	15	100%	14	93.3%	29	96.7%	0.146
	Yes	0	0.0%	1	6.7%	1	3.3%	
Medication	No	5	33.3%	4	26.7%	9	30%	0.431
	Yes	10	66.7%	11	73.3%	21	70%	

1 Chi-Square Test

**Caption:** TMD= temporomandibular disorder N = number of subjects; % = percentage.

**Figure 1:**
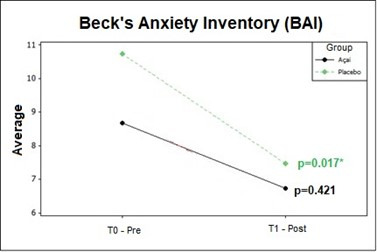
BAI comparison pre and post treatments

The comparison of anxiety symptoms reported in BAI questionnaire before and after the treatments showed significant differences for the group without active (placebo) as shown in Figure 1.

**Caption:** T0- Pre: initial assessment/pre-treatment; T1 – Post: final/post-treatment assessment; *:significant value. Statistics: Kruskal-Wallis Test

When comparing the pre and post-treatment moments for the Tinnitus Handicap Inventory (THI) questionnaire, significant differences were observed for the açaí group (Table 2).

**Table 2:** Pre and post-treatment comparison for THI.

		**Mean**	**Median**	**Standard Deviation**	**CV**	**Min**	**Max**	**N**	**CI**	**P-value**
Açaí	Pre	37.6	30	24.8	66%	6	78	15	12.6	0.006*
	Post	27.6	22	18.4	67%	6	68	15	9.3	
Placebo	Pre	41.2	44	19.1	46%	12	84	15	9.7	0.093
	Post	32.4	24	22.7	70%	6	86	15	11.5	

* = significant p-value

**Caption:** THI= tinnitus handicap inventory; CV = coefficient of variation; Min = minimum; Max = maximum, N = number of subjects; CI = confidence interval. Statistics^1^: Kruskal-Wallis Test

**Figure 2:**
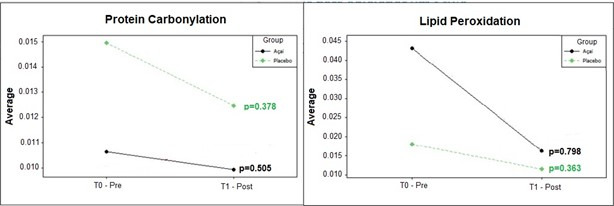
Pre and post-treatment oxidative stress biomarkers

Next, the effect of antioxidant supplementation, by açaí extract, on oxidative stress biomarkers was evaluated, comparing the pre and post-treatment moments (Figure 2).

**Caption:** Statistics: Kruskal-Wallis Test; T0- Pre: initial assessment/pre-treatment; T1 – Post: final/post-treatment assessment

## DISCUSSION

Tinnitus is considered the third worst symptom for human beings, being surpassed only by intense and intractable pain and dizziness. It is believed that about 27.5% of the world population lives with this symptom, which makes it a public health problem. It is a disorder that produces extreme discomfort, difficult to characterize and treat due to its high degree of multifactoriality, which causes drastic changes in the lives of those who perceive it^(20)^.

There is a prevalence of 81.8% of the use of medication for psychological disorders among the public with tinnitus^(21,22)^ and, it is directly proportional to the increase in terms of age, with clonazepam and fluoxetine being the most used in a sample of 1.159 individuals^(22)^. In common agreement, in this research, 70% of the total sample make use of controlled medications and, when asked about feeling anxious or depressed, 63.3% indicated some degree of anxiety (Table 1).

Anxiety symptoms can influence auditory sensitivity and change sound perception, sharpening some of them to the point of becoming a potential threat^(23)^. The relationship between tinnitus and anxiety is strongly studied in the literature, demonstrating a link between emotional factors and the increased severity of tinnitus^(24)^. Thus, the higher the THI grade, the more susceptible the individual with tinnitus will be to emotional changes. Furthermore, tinnitus discomfort depends on other factors such as cognition and personality traits^(25)^.

In the present study, there seems to be a relationship between the severity of tinnitus and psychological aspects, more specifically, anxiety symptoms, as the placebo group presented higher means in BAI questionnaire, corresponding to a mild to moderate degree of anxiety (Figure 1), in addition to higher THI scores (Table 2) which demonstrates greater sensitivity or influence of one symptom on the other.

After three months of intervention, the placebo group showed statistically significant differences (p-value = 0.017) for anxiety symptoms (Figure 1), this demonstrates that passive reception and capsules without active compounds influenced the results for this group due to the psychological mechanisms involved, for example, personal motivation, bond established between therapist and volunteer, by frequent meetings, which can generate an increase in positive expectations about the treatment^(26)^. Therefore, it is one more indication that the placebo effect is a beneficial result of a treatment, which arises from the positive expectations of the patient due to the fact he is being treated, much more than the treatment itself.

Different antioxidant supplements (Ginkgo biloba, lipoic acid, papaverine hydrochloride, vitamin C and E, among others) have already been tested as a treatment for tinnitus, recently, randomized, double-blind, placebo-controlled clinical trials showed efficacy in supplementation of different compounds (vitamins, minerals, phytochemicals and alpha lipoic acid) after treatment for three months. The authors^(6)^ found a reduction in THI scores (p= 0.015) in individuals with and without hearing loss and a mean age of 56.5 years. The present research corroborates these findings, even though other compound was used, it was possible to observe a reduction in THI scores (Table 2) in adult individuals, with a mean age of 50.5 years, with chronic tinnitus after three months of treatment.

Another study with the supplementation of several compounds (Ginkgo biloba, lipoic acid, papaverine hydrochloride, vitamin C and E), in 58 elderly people with clinical complaints of tinnitus associated with hearing loss, obtained THI score equal to six before and after treatment months^(25)^. The exclusion of individuals who had some metabolic comorbidity and other otological complaints was observed in the two studies mentioned above^(6,25)^, in contrast to this study, which did not exclude any comorbidity. Furthermore, the study^(25)^ with several compounds contemplated na older population and with different degrees of hearing loss, which may have influenced the failure of the proposed treatments.

There is a generalization in the eligibility criteria when the topic is treatment with antioxidant supplements^(5,6,25,27)^, there is the inclusion of healthy individuals, with or without sensorineural hearing loss, and idiopathic etiology of tinnitus, on the other hand individuals with metabolic diseases, make use of controlled medications and present other otological etiologies, for example, dizziness, are excluded from the samples. However, in the present study, all the criteria mentioned were considered and the results demonstrate that supplementation with açaí extract is beneficial regardless of the underlying etiology (Table 1).

In relation to biomarkers, despite the fundamental role of oxidative stress in the pathogenesis of tinnitus, data on the effectiveness of antioxidant supplementation in oxidative stress biomarkers in tinnitus are rare^(5)^. A recent study on the effect of cranial osteopathy in 28 individuals with tinnitus showed a significant increase (p<0.03) in protein carbonylation after treatment, which may be the result of osteopathy as an agent of physical and oxidative stress, after a single session, however, there were no changes in biomarkers for the substances that are reactive to the thiobarbituric acid^(7)^.

In the present study, there were no significant differences between pre and post-treatment moments for oxidative stress biomarkers (Figure 2), however, individuals reported a decrease of the perception and discomfort regarding tinnitus. This finding corroborates another study^(6)^ on antioxidant supplementation, which evaluated 35 individuals with a mean age of 56.5 years, in which there was symptom improvement, but without significant changes in oxidative damage.

Higher plasma concentrations of oxidative stress biomarkers and lower antioxidant activity have been reported in individuals with tinnitus in comparison with healthy individuals^(5)^. However, research data on the effectiveness of antioxidant supplementation on tinnitus are limited and conflicting, and oxidative stress biomarkers have not been evaluated in most studies. The present study arises as a possibility of including the açaí extract in tinnitus control, as an oral, natural and external antioxidant agent, with the objective of reaching the balance of the oxidative metabolism.

In a pilot study with 14 individuals, it was verified that the daily consumption of juice containing açaí decreased lipid peroxidation in people with chronic joint disease, reducing pain in 12 weeks significantly^(27)^. When comparing tinnitus and chronic pain, both present different causes, can be influenced by the CNS, modulate its intensity over time and have a strong psychological component^(27)^.

Lipid peroxidation is a cascade of biochemical events that are highly toxic to the the body, in such a way that there is a loss of cell membrane selectivity, for the entry of nutrients and the exit of toxic substances, which can generate several pathologies. This selectivity imbalance causes an increase in oxidative stress, leading to damage in lipids, proteins and also in cellular deoxyribonucleic acid (DNA)^(28)^, thus, the use of oral antioxidants is aimed at the lost cellular balance.

Therefore, it is believed that lipid peroxidation needs to be better studied in the involvement of antioxidants and chronic tinnitus. Also, as a limitation of this study, there was a small number of individuals in each group, compromising the identification of differences between means, with statistical significance, between the variables. However, it is believed that there is a relationship between the discomfort caused by tinnitus, anxiety symptoms and levels of oxidative stress, once the values ​​before and after treatment were lower.

Furthermore, despite advances in the search for different treatments for the symptom, it is relevant that natural proposals, without contraindications, receive greater attention in the scientific world.

## CONCLUSION

Oral antioxidant supplementation, consisting of açaí extract, showed favorable effects on tinnitus, reducing discomfort with the symptom, regardless of the underlying etiology, being, then, considered a treatment modality. However, the effect of this supplementation on anxiety symptoms and oxidative stress biomarkers needs further investigation.
