# Supplementary material for: Supplementation with açaí (Euterpe Oleracea Martius) for the treatment of chronic tinnitus: effects on perception, anxiety levels and oxidative metabolism biomarkers
Source: Codas. 2022 Jan 26;34(4):e20210076. doi: 10.1590/2317-1782/20212021076 (PMC9886123; doi:10.1590/2317-1782/20212021076)
Supplement: Translation

# Translation: Suplementação com açaí (Euterpe Oleracea Martius) para o tratamento do zumbido crônico: efeitos na percepção, níveis de ansiedade e biomarcadores de metabolismo oxidativo

**Authors:** Sheila Jacques Oppitz, Michele Vargas Garcia, Rúbia Soares Bruno, Cleide Monteiro Zemolin, Bruna Olegário Baptista, Bárbara Osmarin Turra, Fernanda Barbisan, Ivana Beatrice Mânica da Cruz, Aron Ferreira da Silveira

**Affiliations:** 1 Programa de Pós-graduação em Distúrbios da Comunicação Humana, Universidade Federal de Santa Maria – UFSM - Santa Maria (RS), Brasil.; 2 Programa de Pós-graduação em Gerontologia, Universidade Federal de Santa Maria – UFSM - Santa Maria (RS), Brasil.; 3 Hospital Universitário de Santa Maria, Universidade Federal de Santa Maria – UFSM - Santa Maria (RS), Brasil.; 4 Departamento de Morfologia, Universidade Federal de Santa Maria – UFSM - Santa Maria (RS), Brasil.

**Keywords:** Audição, Zumbido, Ansiedade, Euterpe, Antioxidantes, Estresse Oxidativo, Efeito Placebo

## Abstract

**Objetivo:**

Investigar os efeitos da suplementação antioxidante com extrato de açaí no incômodo com o zumbido crônico e a relação com os níveis de ansiedade e metabolismo oxidativo, não excluindo a sobreposição de enfermidades.

**Método:**

Ensaio clínico, randomizado, controlado por placebo. Participaram 30 indivíduos, com média de 50,5 anos, 14 do sexo masculino e 16 do feminino, com limiares auditivos normais ou perda auditiva sensorioneural até grau leve bilateralmente, divididos em dois grupos: Grupo Placebo (sem ativo) e Grupo Açaí (100mg de extrato de açaí). Aplicaram-se os seguintes procedimentos antes e após três meses dos tratamentos: Tinnitus Handicap Inventory (THI), Inventário de Ansiedade de Beck (BAI) e amostras de sangue para avaliação de biomarcadores de estresse oxidativo (Peroxidação Lipídica e Carbonilação de proteínas).

**Resultados:**

Houve redução do incômodo do zumbido para o grupo açaí, verificado por meio do THI (p=0,006). Diferenças significativas foram constatadas na pontuação dos sintomas comuns para os quadros de ansiedade no grupo placebo (p=0,016) porém, o mesmo não foi observado para os biomarcadores de metabolismo oxidativo, apesar de haver uma diminuição dos valores pós-tratamento para os grupos.

**Conclusão:**

A suplementação antioxidante oral, com extrato de açaí, manifestou efeitos favoráveis no zumbido, reduzindo o desconforto com o sintoma, independente da etiologia de base, podendo ser considerada uma modalidade de tratamento. Entretanto, o efeito dessa suplementação nos sintomas de ansiedade e em biomarcadores de estresse oxidativo precisa de maior investigação.

## INTRODUÇÃO

O açaí (Euterpe oleracea Mart.) é uma fruta típica do norte do Brasil recentemente popularizada por sua alta capacidade antioxidante relacionada à presença de ácidos fenólicos, flavonoides e antocianinas^(1)^. Atualmente, diferentes estudos têm sido realizados com essa fruta, como nos casos de diabetes, dislipidemia, doença cardiovascular, efeito anti-hipertensivo^(2)^, terapia alternativa para doenças neuropsiquiátricas^(3)^, entre outras.

Antioxidantes são compostos que atuam inibindo e/ou diminuindo os efeitos do estresse oxidativo, desencadeados pelos radicais livres e compostos oxidantes. São importantes porque, com o combate aos processos oxidativos, há menores danos ao Ácido Desoxirribonucleico (DNA) e às macromoléculas, amenizando, assim, os danos cumulativos que podem desencadear diferentes doenças^(4)^.

A natureza do dano celular provocado pelas Espécies Reativas de Oxigênio (EROs) depende do seu local de formação, no entanto, as principais moléculas suscetíveis a danos induzidos por estresse oxidativo são os lipídios de membrana, onde há destruição de sua integridade, provocando morte ou apoptose celular. Quando há aumento na formação de EROs no Órgão de Córti, resulta em dano epitelial sensorial permanente^(5)^.

Quanto ao zumbido, há relatos de maiores concentrações plasmáticas nos biomarcadores de estresse oxidativo e baixa atividade antioxidante, fundamentando o papel do status oxidativo na patogênese do zumbido. No entanto, o uso de biomarcadores de estresse oxidativo para comparar antes e após tratamentos são escassas, estudos atuais com biomarcadores verificam melhora sintomatológica, mas pouca ou nenhuma diferença para o status oxidativo^(6,7)^.

De acordo com a literatura, a terapia antioxidante em pacientes com zumbido idiopático pode reduzir o estresse oxidativo e danos no ouvido interno, reduzindo também, a intensidade e desconforto do zumbido. O suplemento antioxidante pode ser uma opção adicional de terapia para pacientes com zumbido, no entanto, estudos clínicos são necessários para determinar o papel protetor dos antioxidantes e escolher o protocolo terapêutico mais adequado^(8)^.

O zumbido é considerado um problema de saúde que gera impacto econômico. Uma pesquisa mostrou que gastos em saúde de pacientes com queixa de zumbido são elevados, pois, de modo geral, este grupo busca ajuda de vários profissionais. Além disso, os autores destacaram que os custos com cuidados de saúde estão diretamente proporcionais à gravidade do sintoma^(9)^.

Atualmente, não há um único tratamento que seja efetivo para todos os pacientes. A heterogeneidade do zumbido é um desafio adicional à pesquisa clínica, visto que o sintoma pode diferir em muitos aspectos, como localização, características sonoras, causas subjacentes, condições comórbidas, entre outros^(10)^.

Embora haja avanços em estudos específicos sobre o zumbido, sua fisiopatologia e, consequentemente, etiologia ainda não foram completamente esclarecidas. Aliado à subjetividade desta manifestação, mais a sobreposição das enfermidades e dos sintomas que geralmente acometem esses pacientes, dificulta a obtenção de um bom resultado terapêutico^(11)^. Entretanto, buscar novas medidas que contemplem o indivíduo como um todo e auxiliem na redução do zumbido torna-se necessário. Salienta-se ainda, que devido a multifatoriedade do sintoma, muitas vezes, o tratamento precisa ser combinado e não isolado.

Desta forma, o extrato do açaí apresenta um atraente perfil farmacológico, antineoplásico e antioxidante com propriedades sem efeitos secundários, o que sugere a sua utilização em terapia de prevenção ou tratamento de sintomas e doenças^(12)^.

Portanto, alimentos naturais, como o extrato de açaí, poderiam ajudar sujeitos com zumbido crônico a diminuir o incômodo com o sintoma. Com isso, o objetivo deste estudo foi investigar os efeitos da suplementação antioxidante com extrato de açaí no incômodo com o zumbido crônico e a relação com os níveis de ansiedade e metabolismo oxidativo, não excluindo a sobreposição de enfermidades.

## MÉTODO

Estudo duplo-cego, de abordagem qualitativa e quantitativa, desenvolvido em forma de ensaio clínico randomizado, de caráter descritivo com delineamento transversal e longitudinal. A amostra foi composta por conveniência e os atendimentos foram realizados em um Ambulatório de Audiologia Clínica de uma Instituição Pública de Ensino Superior.

### Aspectos éticos

Aprovado pelo Comitê de Ética em Pesquisa sob nº CAAE: 96740718.4.0000.5346, com número do Registro Brasileiro de Ensaios Clínicos (ReBEC): RBR-8z4mhq. Os indivíduos que concordaram em participar desta pesquisa assinaram o Termo de Consentimento Livre e Esclarecido elaborado de acordo com a Resolução 466/12, do Conselho Nacional de Saúde.

### Critérios de elegibilidade

Para participar do estudo, os indivíduos deveriam ter idade igual ou superior a 18 anos; Queixa de zumbido crônico (percepção mínima de seis meses) unilateral ou bilateral; Limiares auditivos dentro dos padrões de normalidade bilateralmente^(13)^ ou perda auditiva sensorioneural de grau até leve na média quadritonal (500, 1000, 2000 e 4000Hz)^(13)^; Nota de incômodo de no mínimo quatro na Escala Visual Analógica, considerado um incômodo moderado do sintoma^(14)^.

Além disso, os mesmos não poderiam, no período da participação deste estudo, ter apresentado sintomas e/ou diagnóstico de comprometimento de orelha média, ter iniciado novo tratamento (farmacológico ou terapêutico) ou ter sido diagnosticado com alguma doença de qualquer origem. Também, não poderiam fazer uso de aparelhos de amplificação sonora individual (AASI) para que não houvesse interferência nos achados do presente estudo.

### Caracterização da amostra

Inicialmente foram convidados a participar desse estudo, 62 indivíduos, sendo que destes, oito não compareceram à consulta, cinco já haviam feito tratamento anterior para o sintoma e cinco apresentaram perda auditiva sensorioneural de grau maior que moderado. Além do mais, quatro referiram nota Escala Visual Analógica (EVA) inferior a três, uma era gestante, nove não concluíram os três meses de tratamento, totalizando uma amostra de 30 voluntários, 15 em cada grupo, com média de idade de 50,5 anos, 14 do gênero masculino e 16 do feminino.

Os indivíduos foram alocados em dois grupos diferentes e receberam, um dos seguintes esquemas terapêuticos: O placebo foi o grupo controle, sem ativo, disponibilizado por meio de cápsulas de amido e o extrato de açaí foi o grupo que recebeu o tratamento com administração de extrato de açaí seco (100mg/cápsula).

A randomização foi realizada para que cada voluntário da pesquisa pudesse ter a mesma probabilidade de ser alocado nos dois grupos do estudo, contribuindo para que as características da amostra fossem homogêneas. Foi realizado no formato de sorteio de cartas, no qual o voluntário retirou a carta correspondente a um dos tratamentos propostos.

O cálculo amostral foi baseado na variação do escore total do questionário THI, adotando-se nível de significância de 5% e poder do teste de 80%, para se detectar uma diferença mínima entre os participantes de 20 pontos no escore total do questionário, estimou-se que fossem necessários ao menos 15 sujeitos, conforme o *software One-way* ANOVA.

Quanto aos procedimentos realizados, segue o detalhamento a seguir:

**Anamnese de Investigação do Zumbido:** Anamnese baseada no *Clinical Practice Guideline: Tinnitus* ^(15)^, contendo informações sobre histórico clínico-médico, hábitos, e questões específicas sobre zumbido (localização, frequência, intensidade, tempo de percepção, entre outros). Salienta-se que todas as comorbidades foram consideradas por meio do relato do indivíduo.
***Tinnitus Handicap Inventory* – THI:** Instrumento traduzido e validado para o português brasileiro, composto por 25 perguntas e com três alternativas de resposta: sim (4 pontos), às vezes (2 pontos) e não (0 pontos). A pontuação pode variar de 0 (zero) a 100 pontos. Assim, pontuações entre 0 e 16 indicam grau de incômodo ligeiro, entre 18 e 36, incômodo leve, entre 38 e 56, moderado, entre 58 e 76, incômodo intenso e, entre 78 e 100, incômodo catastrófico. O questionário foi aplicado em forma de entrevista individual a fim de assegurar o preenchimento completo e a compreensão da questão pelo indivíduo pesquisado^(16)^.
**Inventário de Ansiedade de Beck - *Beck Anxiety Inventory* – BAI:** Esse inventário foi adaptado por Cunha (2001)^(17)^, apresentando bons coeficientes de fidedignidade e validade. A escala consiste de 21 itens descrevendo sintomas comuns em quadros de ansiedade, a resposta consta dentro de uma escala de quatro pontos: Absolutamente não; Levemente; Moderadamente; Gravemente. Os itens somados resultam em escore total que pode variar de zero a 63, gerando o nível de ansiedade: 0 – 7 pontos = nível mínimo de ansiedade; 8 – 15 pontos = leve ansiedade; 16-25 pontos = ansiedade moderada; 26-63 pontos = ansiedade severa.
**Avaliação do metabolismo oxidativo:** Após a finalização das avaliações auditivas, coletou-se amostras de sangue periférico (20mL) do braço do voluntário, utilizando tubos top *Vacutainer* ® (*BD Diagnostics, Plymouth, Reino Unido*), com anticoagulante Ácido etilenodiaminotetracético (EDTA). A coleta foi realizada na avaliação inicial e final por profissional enfermeiro com experiência na coleta sanguínea.

Inicialmente, as amostras foram centrifugadas a 2000 rotações por minuto (rpm) por 10 minutos, para que ocorresse a separação do plasma, glóbulos brancos e eritrócitos. A série branca do sangue foi acondicionada em tubos com *Ficoll-Histopaque- Sigma* (*Ficoll-Paque Plus - GE HealthCare*, densidade 1,077), ocorrendo a centrifugação, separação, lavagem e acondicionadas em tubos com trizol para análises posteriores. O plasma foi acondicionado em microtubos e armazenado a -80ºC para avaliações seguintes.

Os biomarcadores de estresse oxidativo não possuem critérios de normalidade na literatura, o que se sabe é que quanto maior os valores, maior é o dano, portanto, os indivíduos devem ser auto comparados antes e após o tratamento. Com o plasma foram realizadas as seguintes análises:

Peroxidação Lipídica (Lipoperoxidação) - Ensaio TBARS (Substâncias Reativas ao Ácido Tiobarbitúrico): A Lipoperoxidação (LPO) pode ser definida como uma cascata de eventos bioquímicos resultante da ação dos Radicais Livres (RL) sobre os lipídios insaturados das membranas celulares, levando à grave alteração da membrana celular, causando perda da fluidez, alteração da função secretora e dos gradientes iônicos de transmembrana. As alterações nas membranas levam a transtornos da permeabilidade, alterando o fluxo iônico e o fluxo de outras substâncias, o que resulta na perda da seletividade para entrada e/ou saída de nutrientes e substâncias tóxicas à célula, alterações do DNA (Ácido Desoxirribonucleico) e comprometimento dos componentes da matriz extracelular (proteoglicanos, colágeno e elastina).

Portanto, avaliou-se a peroxidação lipídica por meio do ensaio TBARS por meio da reação do ácido tiobarbitúrico com o Malondialdeído (MDA), quanto maior a presença de MDA, maiores os índices de lipoperoxidação. Todos os tratamentos foram acrescidos de água, ácido fosfórico (H_3_PO_4_) a 10%, ácido tricloroacético (TCA) a 20% e ácido tiobarbitúrico (TBA) 1,2%. Para tal foi confeccionada uma curva de dosagem de malondialdeído (MDA) para posterior determinação da equivalência de cada amostra em teste. Após a adição de todos os reagentes, realizou-se uma incubação de uma hora, a 95ºC, e em seguida, com as amostras a temperatura ambiente, foi efetuada a leitura das absorbâncias a 532 nm em espectrofotômetro. Os resultados foram expressos como nmol de MDA/mL de células^(18)^.

Carbonilação de proteínas: Durante o estresse oxidativo em proteínas, ocorre a fragmentação das cadeias e oxidação de quase todos os tipos de aminoácidos com produção frequente de compostos carbonilados do tipo aldeídos, cetonas, amidas, carboxilas e ésteres. Esses danos foram mensurados pela determinação de formação de grupos carbonil baseados na reação com 2,4-dinitrofenil-hidrazina (DNPH), um reagente que reage com as proteínas carboniladas como previamente descrito por Morabito et al.^(19)^. O conteúdo de formação de carbonil é determinado por meio de leitura em leitor de placas, em placa de ELISA UV no comprimento de onda 370nm.

Portanto, para o desfecho primário tem-se a necessidade de observar a melhora do incômodo com o zumbido na vida dos indivíduos por meio da diminuição dos escores do THI, de forma que o desfecho secundário se baseie também na diminuição dos escores, mas para a ansiedade (BAI) e biomarcadores de metabolismo oxidativo (TBARS e carbonilação).

### Extrato de açaí (*Euterpe Oleracea Martius*) como forma de tratamento experimental

O fornecimento do extrato de açaí foi por meio de cápsulas devidamente patenteadas e obtidas pela empresa PHARMANOSTRA, com certificado de análise e Registro pela Agência Nacional de Vigilância Sanitária (ANVISA) nº 669820036. Sendo preservadas as características químicas e farmacológicas, garantindo a sua ação biológica, a segurança de utilização e a valorização de seu potencial terapêutico.

A posologia utilizada de extrato de açaí foi 100mg diários (uma cápsula diária) da polpa da fruta. Um estudo piloto testou a dosagem por meio da comparação de 50mg de açaí em cápsulas; 100mg; e 500mg ou mais (automedicação), mostrando-se mais eficaz com 100mg. Os sujeitos que se automedicaram com 500mg ou mais, apresentaram como efeito adverso cefaleia tensional de intensidade moderada diariamente que, no prazo de dois dias após a diminuição para 100 miligramas, a cefaleia desapareceu, mas o zumbido continuou. Portanto, A dosagem do açaí precisa ser monitorada, de forma cuidadosa e periódica, no sentido de evitar a automedicação equivocada, havendo um limite do extrato que o define como natural ou tóxico para o organismo, o qual pode resultar em cefaleia tensional de intensidade moderada e aumento da percepção do zumbido.

Todos os voluntários foram orientados a tomar uma cápsula por dia, no mesmo horário, preferencialmente com água, durante três meses, duração mínima para que ocorra mudanças significativas em relação ao metabolismo oxidativo. Houve o retorno dos indivíduos mensalmente para retirada das cápsulas junto à pesquisadora. As substâncias utilizadas no tratamento, não foram identificadas nominalmente nos frascos em que foram acondicionadas, mas sim com símbolos definidos por um indivíduo externo, como forma de cegamento dos pesquisadores e voluntários.

Vale ressaltar que, no presente estudo não houve ocorrência de efeitos colaterais ou adversos com o uso das substâncias durante o tempo e na dose empregada (100mg). Sendo esse, um dos fatores relevantes a serem levados em consideração durante um tratamento.

Após o tratamento experimental, os indivíduos foram submetidos a uma reavaliação composta pelos mesmos procedimentos da primeira avaliação, elencados anteriormente. Além disso, foi oferecido Aconselhamento Fonoaudiológico, após a reavaliação, assim como os encaminhamentos aos profissionais quando necessário.

Os dados foram registrados no Microsoft Office Excel (2010) e a distribuição da normalidade foi determinada pelo teste paramétrico Kolmogorov-Smirnov. Para o cálculo de hipóteses para variáveis nominais independentes foi utilizado o teste Qui- Quadrado. As variáveis normalmente distribuídas foram avaliadas por meio do teste Kruskal-Wallis. O nível de significância aceito foi de p< 0,05 (5%) com intervalos de confiança construídos com 95%.

## RESULTADOS

No que tange às características da amostra, com relação à audição, mostraram-se os mais prevalentes, perda auditiva do tipo sensorioneural em frequências agudas (55,6%), limiares auditivos dentro dos padrões de normalidade (33,3%) e perda auditiva sensorioneural leve (11,1%).

No que se refere ao zumbido, o tipo mais encontrado foi cigarra com manifestação contínua, as medidas psicoacústicas predominantes foram: frequência de 6000 Hz e intensidade de 15 dB.

A presença de algumas comorbidades podem estar relacionados ao incômodo e permanência do zumbido (Tabela 1).

**Tabela 1:** Comparação de comorbidades entre os grupos

		Açaí		Placebo		Total		P-valor^1^
		N	%	N	%	N	%	
DTM	Não	10	66,7%	7	46,7%	17	56,7%	0,293
	Sim	5	33,3%	8	53,3%	13	43,3%	
Tontura	Não	10	66,7%	14	93,3%	24	80%	0,146
	Sim	5	33,3%	1	6,7%	6	20%	
Psicológico	Não	6	40,0%	5	33,3%	11	36,7%	0,741
	Sim	9	60,0%	10	66,7%	19	63,3%	
Hipercolesterolemia	Não	11	73,3%	11	73,3%	22	73,3%	0,887
	Sim	4	26,7%	4	26,7%	8	26,7%	
Hipertensão	Não	10	66,7%	12	80,0%	22	73,3%	0,484
	Sim	5	33,3%	3	20,0%	8	26,7%	
Hormonal	Não	15	100%	14	93,3%	29	96,7%	0,146
	Sim	0	0,0%	1	6,7%	1	3,3%	
Medicamentos	Não	5	33,3%	4	26,7%	9	30%	0,431
	Sim	10	66,7%	11	73,3%	21	70%	

1 Test Qui-Quadrado

**Legenda:** DTM= disfunção temporomandibular; N = número de sujeitos; % = porcentagem.

A comparação dos sintomas de ansiedade referidos por meio do questionário BAI antes e após os tratamentos demonstrou diferenças significativas para o grupo sem ativo (placebo) conforme demonstra a Figura 1.

**Figura 1:** Comparação do BAI antes e após os tratamentos

**Legenda:** T0- Pré: avaliação inicial/pré-tratamento; T1 – Pós: avaliação final/pós-tratamento; *:valor significativo. Estatística: Teste Kruskal-Wallis

Na comparação dos momentos pré e pós-tratamento para o questionário Tinnitus Handicap Inventory (THI) pode-se observar diferenças significativas para o grupo com açaí (Tabela 2).

**Tabela 2:** Comparação pré e pós-tratamento para THI

		Média	Mediana	Desvio Padrão	CV	Min	Max	N	IC	P-valor
Açaí	Pré	37,6	30	24,8	66%	6	78	15	12,6	**0,006** *
	Pós	27,6	22	18,4	67%	6	68	15	9,3	
Placebo	Pré	41,2	44	19,1	46%	12	84	15	9,7	0,093
	Pós	32,4	24	22,7	70%	6	86	15	11,5	

* = p-valor significativo

**Legenda:** THI= tinnitus handicap inventory; CV= coeficiente de variação; Mín = mínimo; Máx = máximo, N = número de sujeitos; IC = intervalo de confiança. Estatística^1^: Teste Kruskal-Wallis

A seguir, avaliou-se o efeito da suplementação antioxidante, por extrato de açaí, em biomarcadores de estresse oxidativo, comparando os momentos pré e pós-tratamento (Figura 2).

**Figura 2:** Biomarcadores de estresse oxidativo pré e pós-tratamento

**Legenda:** TBARS = substâncias reativas ao ácido tiobarbitúrico. Estatística: Teste Kruskal-Wallis; T0- Pré: avaliação inicial/pré-tratamento; T1 – Pós: avaliação final/pós-tratamento

## DISCUSSÃO

O zumbido é considerado o terceiro pior sintoma para o ser humano, sendo superado apenas pelas dores e tonturas intensas e intratáveis. Acredita-se que cerca de 27,5% da população mundial conviva com esse sintoma, o que o torna um problema de saúde pública. É um transtorno que produz extremo desconforto, de difícil caracterização e tratamento devido ao seu alto grau de multifatoriedade, que causa mudanças drásticas na vida de quem o percebe^(20)^.

Há uma prevalência de 81,8% do uso de medicamentos para transtornos psicológicos entre o público com queixa de zumbido^(21,22)^ e, é diretamente proporcional ao aumento da idade, sendo o clonazepam e a fluoxetina com maior uso em uma amostra de 1.159 indivíduos^(22)^. Em comum acordo, na presente pesquisa, 70% da amostra total faz uso de medicamentos controlados e ao serem questionados sobre achar-se ansioso ou depressivo 63,3% indicaram algum grau de ansiedade (Tabela 1).

Os sintomas de ansiedade podem influenciar na sensibilidade auditiva e alterar a percepção do som, aguçando alguns deles, a ponto de se tornarem uma ameaça em potencial^(23)^. A relação entre zumbido e ansiedade é fortemente estudada na literatura, demonstrando uma ligação entre os fatores emocionais e o aumento da severidade do zumbido^(24)^. Desta forma, quanto maior for o grau no THI, mais susceptível o indivíduo com zumbido estará para alterações emocionais. Além do mais, o incômodo com o zumbido depende de outros fatores como cognição e traços de personalidade^(25)^.

No presente estudo, parece existir uma relação entre a gravidade do zumbido com aspectos psicológicos, mais especificamente, os sintomas ansiosos, pois o grupo placebo apresentou maiores médias no questionário BAI, correspondendo a um grau leve a moderado de ansiedade (Figura 1), além de maiores escores de THI (Tabela 2) demonstrando maior sensibilidade ou influência de um sintoma sobre o outro.

Após os três meses de intervenção o grupo placebo, apresentou diferenças estatisticamente significantes (p-valor = 0,017) para os sintomas ansiosos (Figura 1), isso demonstra que o acolhimento passivo e as cápsulas sem compostos ativos, influenciaram nos resultados para esse grupo devido aos mecanismos psicológicos envolvidos, por exemplo, motivação pessoal, vínculo estabelecido entre terapeuta e voluntário, pelos encontros frequentes, podendo gerar um aumento nas expectativas positivas sobre o tratamento^(26)^. Portanto, é mais um indicativo de que o efeito placebo é um resultado benéfico de um tratamento, que surge a partir de expectativas positivas do paciente de estar sendo tratado, muito mais do que o próprio tratamento em si.

Diferentes suplementações antioxidantes (*Ginkgo biloba*, ácido lipóico, cloridrato de papaverina, vitamina C e E, entre outros) já foram testadas como tratamento para o zumbido, recentemente, ensaios clínicos, randomizados, duplo-cegos e controlado por placebos, mostraram eficácia na suplementação de diferentes compostos (vitaminas, minerais, fitoquímicos e ácido alfa-lipóico) após tratamento por três meses. Os autores^(6)^ encontraram redução nos escores de THI (p= 0.015) em indivíduos com e sem perda auditiva e idade média de 56,5 anos. A presente pesquisa corrobora com os achados referidos, apesar de utilizar outro composto, foi possível observar a redução nos escores do THI (Tabela 2) em indivíduos adultos, com média de idade de 50,5 anos, com zumbido crônico após três meses de tratamento.

Outro estudo com a suplementação de vários compostos (*Ginkgo biloba*, ácido lipóico, cloridrato de papaverina, vitamina C e E), em 58 idosos com queixa clínica de zumbido associado à perda auditiva, obteve escore de THI igual antes e após o tratamento de seis meses^(25)^. Observou-se a exclusão dos indivíduos que tiveram alguma comorbidade metabólica e outras queixas otológicas nos dois estudos citados anteriormente^(6,25)^, em contrapartida a este estudo, que não fez exclusão de nenhuma comorbidade. Além do mais, o estudo^(25)^ com vários compostos contemplou uma população mais idosa e com diferentes graus de perda auditiva, o que pode ter influenciado no insucesso dos tratamentos propostos.

Nota-se uma generalização nos critérios de elegibilidade quando o assunto é tratamento com suplementos antioxidantes^(5,6,25,27)^, há a inclusão de indivíduos saudáveis, com ou sem perda auditiva sensorioneural, e etiologia idiopática do zumbido, já os indivíduos que apresentam doenças metabólicas, usam medicamentos controlados e possuem outras etiologias otológicas, por exemplo, a tontura, são excluídos das amostras. Entretanto, no presente estudo, todos os critérios citados foram considerados e os resultados demonstram que a suplementação com extrato de açaí é benéfica independente da etiologia de base (Tabela 1).

Em relação aos biomarcadores, apesar do papel fundamental do estresse oxidativo na patogênese do zumbido, dados sobre a eficácia da suplementação antioxidante nos biomarcadores de estresse oxidativo no zumbido são escassos^(5)^. Um estudo recente, sobre o efeito da osteopatia craniana, em 28 indivíduos com zumbido, demonstrou um aumento significativo (*p*<0,03) da carbonilação de proteínas após o tratamento, podendo ser o resultado da osteopatia como um agente de estresse físico e oxidativo, após uma única sessão, no entanto, não houve mudanças nos biomarcadores para as substâncias reativas ao ácido tiobarbitúrico^(7)^.

No presente estudo, não houve diferenças significativas entre os momentos pré e pós-tratamento para os biomarcadores de estresse oxidativo (Figura 2), no entanto os indivíduos referiram diminuição da percepção e do incômodo do zumbido. Tal achado corrobora com outro estudo^(6)^ de suplementação antioxidante, que avaliou 35 indivíduos com idade média de 56,5 anos, no qual houve melhora sintomatológica, porém sem mudanças significativas de danos oxidativos.

Concentrações plasmáticas mais altas de biomarcadores do estresse oxidativo e menor atividade antioxidante foram relatadas em indivíduos com zumbido em comparação com indivíduos saudáveis^(5)^. No entanto, dados de pesquisa sobre a eficácia da suplementação antioxidante no zumbido são limitadas e conflitantes, além disso, biomarcadores de estresse oxidativo não foram avaliados na maioria dos estudos. O presente estudo surge como possibilidade de inclusão do extrato de açaí no controle do zumbido, como sendo um agente antioxidante oral, natural e externo, com o objetivo de alcançar o equilíbrio do metabolismo oxidativo.

Em estudo piloto com 14 indivíduos verificou que o consumo diário de suco contendo açaí diminuiu a peroxidação lipídica de pessoas com doença articular crônica reduzindo significativamente a dor em 12 semanas^(27)^. Ao compararmos o zumbido e a dor crônica, ambos apresentam causas diversas, podem ser influenciados pelo SNC, modulam sua intensidade ao longo do tempo e possuem forte componente psicológico^(27)^.

A peroxidação lipídica é uma cascata de eventos bioquímicos altamente tóxicos ao organismo, de forma que há perda da seletividade da membrana celular, para entrada de nutrientes e saída de substâncias tóxicas, o que pode gerar diversas patologias. Esse desequilíbrio de seletividade provoca aumento no estresse oxidativo, levando a danos em lipídios, proteínas e no ácido desoxirribonucleico (DNA) celular^(28)^, deste modo, o uso de antioxidantes orais visam ao equilíbrio celular perdido.

Portanto, acredita-se que a peroxidação lipídica precisa ser melhor estudada no envolvimento de antioxidantes e o zumbido crônico. Ainda, como limitação deste estudo, houve um pequeno número de indivíduos em cada grupo, comprometendo a identificação de diferenças entre médias, com significância estatística, entre as variáveis. Todavia, acredita-se que há uma relação entre o incômodo causada pelo zumbido, sintomas ansiosos e níveis de estresse oxidativo, pois os valores antes e pós-tratamento mostraram-se menores.

Além disso, apesar dos avanços em busca de diferentes tratamentos para o sintoma, é relevante que propostas naturais, sem contraindicação, tenham uma atenção maior frente ao mundo científico.

## CONCLUSÃO

A suplementação antioxidante oral, composta por extrato de açaí, manifestou efeitos favoráveis no zumbido, reduzindo o desconforto com o sintoma, independente da etiologia de base, podendo ser considerada uma modalidade de tratamento. Entretanto, o efeito dessa suplementação nos sintomas de ansiedade e em biomarcadores de estresse oxidativo precisa de maior investigação.
